# Effects of masculinity vs. femininity on competence judgement of politician faces and election outcome prediction

**DOI:** 10.1038/s41598-023-44159-7

**Published:** 2023-10-06

**Authors:** Olivia S. Cheung, Davit Jintcharadze

**Affiliations:** 1 Department of Psychology, Division of Science, New York University Abu Dhabi, Abu Dhabi, UAE; 2 Center for Brain and Health, NYUAD Research Institute, New York University Abu Dhabi, Abu Dhabi, UAE

**Keywords:** Human behaviour, Psychology

## Abstract

First impressions of politician faces can be effective in predicting election outcomes, based on perceived competence from candidate photographs. However, it remains unclear whether such effects arose from facial features or other non-facial information present in the photographs (e.g. hairstyles, clothes, or poses). In four pre-registered studies, participants completed two tasks in a counter-balanced order: rating competence of individually presented faces and predicting election outcome of each pair of winner and runner-up faces. We examined competence judgment and election outcome prediction on faces from male politicians depicted on original portraits (Experiment 1), or on computer-generated faces with facial features extracted from the portraits (Experiment 2). The faces were then either masculinized or feminized (Experiments 3 and 4). We found that competence ratings were significantly higher for winners than runners-up and that winners were more likely predicted to win the elections than runners-up in all but Experiment 4, where faces of the winners were feminized and faces of the runners-up were masculinized. Regardless of facial feature changes, correlations were found between competence ratings and election outcome prediction. These findings suggest that facial features are critical for evaluating competence and predicting election outcome, and that masculine features may enhance stereotypical leadership impressions.

## Introduction

At a glance, human observers perceive a vast amount of information from faces, including identity, expressions, race, gender, and trait characteristics (e.g. trustworthiness, attractiveness, competence)^1^. Such information is highly functional and versatile for various social interactions. While rapid social attributions from faces are often useful in daily life (e.g. deciding whether to approach or avoid a stranger), personality traits perceived from faces may also be heavily relied on as heuristics to quickly form preferences and inferences as the basis of important decisions, including casting a vote for a political candidate in a democratic election^2,3^. Although information beyond facial characteristics about political candidates is often readily available for voters, consistent evidence has shown that personality trait judgement made based on visual appearance from photographs by naïve participants who had no prior knowledge about the candidates can reliably predict actual voting outcomes in the real-world or hypothetical voting outcomes in laboratory settings^4–7^.

Among various perceived personality traits, competence is the most consistently found to be predictive of election outcomes. In a typical study^6^, photographs of opposing political candidates were simultaneously shown and participants were asked to distinguish the relative difference in perceived competence^4,6,8,9^. Alternatively, participants were asked to provide competence ratings to individually presented photographs of the candidates^10^. Candidates who were rated more competent often received more votes in the elections^4,6,10^. Moreover, when asked to choose the preferred or winning candidates among pairs of candidates, participants’ selections often matched the real-world outcomes and the winners were consistently chosen. These judgments were highly consistent among participants, even when they only had a glimpse of the faces (e.g. 100 ms)^3,4,6^. Longer or unlimited presentation times of the photographs were not necessary, as neither were found to significantly change the choices. Moreover, deliberation of the decisions reduced or minimized the effects^4^. Preference for the winning politicians over the runners-up based on visual cues has been replicated across elections in multiple countries^11–18^ and has been observed not only among adults but also young children^11^.

Since competence is often considered to be one of the highly desirable leadership qualities across various cultures^19^, it may not be surprising that people with facial characteristics that signal high competence are more likely to be selected as leaders. It has been shown that evaluation of facial appearance may be based on stereotypes of gender roles: masculine facial characteristics likely contributed to perceptions of men’s competence and attractiveness^20^, and perceived facial competence tends to be higher for male than female faces^10,21^. Within each gender, faces with highly prototypically masculine characteristics are also rated as competent^3,21,22^, although female faces with counter-stereotype traits (e.g. masculine features) might be evaluated negatively^23^. Nonetheless, gendered facial appearance is found to be related to voting behavior in both past elections in the real-world and hypothetical elections in the laboratory^20,24^.

But to what extent the perception of competence from visual cues is based on facial or non-facial features? If facial features are critical, would manipulations of the facial features of the winning and losing candidates increase or decrease their perceived competence and margin of predicted winning chance? Indeed, there is evidence that changes in facial features can systematically alter perceived personality traits from faces. For instance, data-driven computational models have been developed using a large set of computer-generated synthetic faces to vary perceived trait dimensions^7,25–27^. Such models have also been validated using unfamiliar human faces with standardized external features (e.g. hairstyle or pose^21^), indicating a close relationship between visual characteristics of facial features and perceived personality traits. However, previous studies that examined the relationship between facial traits and election outcomes primarily used photographs of real-world politicians. Apart from visual differences between the facial features of the candidates, the photographs also often contain non-facial features such as hairstyle, pose, or attire. These non-facial visual features could potentially bias trait judgments or choice behaviors of participants^28–30^. Therefore, it is important rule out the influence of non-facial features to understand whether and how intrinsic facial features may affect perception of competence and prediction of actual election winners.

To date, relatively limited research has directly addressed this issue. A previous study extracted facial features of winners and runners-up from several general or presidential elections (e.g. Bush vs. Kerry, Blair vs. Major)^15^. To prevent participants from recognizing the faces, the winner and runner-up faces were morphed with a composite face that was made from unfamiliar faces, though the resulting faces preserved the facial differences of the winner and runner-up faces. Although the new faces appeared unfamiliar to the participants, the hypothetical voting choices were highly correlated with the actual election outcomes. Moreover, using unfamiliar faces with facial features adjusted along the masculinity-femininity continuum, masculinized faces were more likely to be selected as the leaders in a wartime context (though feminized faces were more likely to be selected as the leaders during a peaceful time)^15^. However, it remains unclear how visual characteristics of the original faces of the winners and runners-up, independent of non-facial cues, might affect perception of potential leadership qualities. Moreover, given the potential differences in visual facial cues among the political candidates, it is possible that direct manipulations of masculinity and femininity of the facial features of the candidates could influence perceived competence and predicted election outcomes.

This study directly examined whether and how facial features extracted from original images alone are sufficient for evaluating competence and predicting election outcomes. We chose to use faces from politicians who took part in a range of lesser-known United States elections, including congressional, gubernatorial, and mayoral races, and recruited participants from the United Kingdom who had limited to no prior knowledge about the political candidates. Across four experiments, we examined how the facial characteristics of the candidates were evaluated. In all experiments (Experiments 1 to 4), participants completed two tasks in a counter-balanced order across participants. In the competence judgment task, participants rated the perceived competence of each individually presented face. In the election judgement task, participants selected among each pair of opposing candidates the one who would likely win the election.

As two tasks were included in the 4 Experiments, the presentation order of the tasks was counter-balanced across participants: half of the participants completed the competence judgment task first (Experiments 1A, 2A, 3A, and 4A), and the other half of the participants completed the election judgment task first (Experiments 1B, 2B, 3B, and 4B). Our main analyses between competence ratings and election outcome prediction focused on first impressions, and thus performance of the two tasks was compared across separate groups of participants as they viewed the face images for the first time (e.g. to investigate the difference in competence judgment between the original faces vs. the computerized faces, the competence ratings in Experiment 1A and Experiment 2A were compared; to examine the relationship between competence judgment and election outcome prediction for the original faces, the competence ratings in Experiments 1A vs. the election outcome prediction accuracy in Experiment 1B were used). Nonetheless, performance of the two tasks within participants (e.g. the competence ratings and election outcome accuracy within Experiment 1A) was also reported (Table 1).

**Table 1:** Descriptive and inferential statistics on the competence ratings for the images of the political candidates.

Experiment	Winner	Runner-up	Competence difference	Correct guesses in election outcome	Election success	Correlation between competence & election within group
1A	4.52 (0.073)	4.32 (0.072)	*t*_109_ = 6.68 *p* < 0.001	56.4% (0.90%)	*t*_109_ = 7.09 *p* < 0.001	*r* = 0.825 *p* < 0.001
1B	4.51 (0.057)	4.26 (0.055)	*t*_124_ = 8.45 *p* < 0.001	56.8% (0.89%)	*t*_124_ = 7.58 *p* < 0.001	*r* = 0.846 *p* < 0.001
2A	4.04 (0.061)	3.93 (0.064)	*t*_120_ = 3.74 *p* < 0.001	55.0% (0.82%)	*t*_120_ = 6.10 *p* < 0.001	*r* = 0.914 *p* < 0.001
2B	4.23 (0.055)	4.09 (0.057)	*t*_132_ = 5.07 *p* < 0.001	55.7% (0.75%)	*t*_132_ = 7.56 *p* < 0.001	*r* = 0.924 *p* < 0.001
3A	4.03 (0.058)	3.73 (0.068)	*t*_121_ = 5.93 *p* < 0.001	64.7% (1.28%)	*t*_121_ = 11.5 *p* < 0.001	*r* = 0.847 *p* < 0.001
3B	4.39 (0.051)	3.84 (0.071)	*t*_126_ = 9.41 *p* < 0.001	59.3% (1.15%)	*t*_125_ = 8.07 *p* < 0.001	*r* = 0.857 *p* < 0.001
4A	3.94 (0.059)	4.03 (0.054)	*t*_122_ = − 2.27 *p* = 0.025	50.0% (1.12%)	*t*_122_ = .0427 *p* = 0.518	*r* = 0.944 *p* < 0.001
4B	4.06 (0.054)	4.10 (0.053)	*t*_126_ = − 0.909 *p* = 0.365	51.2% (1.11%)	*t*_126_ = 1.076 *p* = 0.142	*r* = 0.886 *p* < 0.001

Winner: mean competence ratings for the winner faces. Runner-up: mean competence ratings for the runner-up faces.

In Experiment 1, the original candidate photographs were presented. Experiment 2 used face images with facial features extracted from the photographs. The face images were then modified to create masculinized and feminized versions of the candidates. Experiment 3 showed the masculinized images of the winners and the feminized images of the runners-up. Experiment 4 presented the feminized images of the winners and the masculinized images of the runners-up. Replicating previous findings using candidate photographs (e.g. Chiao et al.^10^; Todorov et al.^6^), we expected that Experiment 1 would reveal higher competence ratings for the winners than the runners-up. We also expected that the relative differences in competence ratings between the opposing candidates would positively correlate with the proportion of the winners being selected among the pairs. Importantly, such correlations were expected not only within the same group of participants^10^ but also across different groups of participants^4,6^.

If these results were at least partially due to the differences in facial features among the candidates, independent of any contributions from other information available from the original images, similar results were also expected for Experiment 2. Furthermore, if competence ratings were increased by masculine facial features and were decreased by feminine facial features^10,21^, the relative differences in competence ratings between the winners and the runners-up should be larger in Experiment 3 than Experiment 4. Likewise, the prediction accuracy of the winners based on the face images was also expected to be higher in Experiment 3 than in Experiment 4. Nonetheless, if both perceived competence ratings and election outcome prediction judgments were dependent on the evaluation of facial features, the correlations between the two tasks should remain high in both Experiments 3 and 4.

## Experiments 1 and 2

### Method

#### Participants

A total of 600 White participants who were United Kingdom (UK) nationals participated online via the Prolific platform (www.prolific.io). All participants reported normal or corrected-to-normal vision and were between 18 to 45 years old. All participants had a 100% approval rate on the platform. Of the total sample, 300 participants completed Experiment 1: in Experiment 1A, 150 participants first completed the competence rating task then the election judgment task; in Experiment 1B, 150 participants first completed the election judgment task then the competence rating task. There was a total of 75 female and 75 male participants recruited in each of Experiments 1A and 1B. Similarly, the other 300 participants with the same demographics completed Experiment 2 (150 participants in Experiment 2A who completed the competence judgment task first, and 150 participants in Experiment 2B who completed the election judgment task first). The study was approved by the New York University Abu Dhabi Institutional Review Board. All participants gave informed written consent prior to the experiment. All methods were performed in accordance with the relevant guidelines and regulations.

Following the pre-registered exclusion criteria (i.e. excluding data of participants that met any of the criteria below: extreme outliers with response times below 200 ms or over 5000 ms for more than 10% of the trials in either task; over 15% of consecutive trials with the response key in the competence rating task; below 0.5 standard deviation in the competence ratings), data from 489 participants were included in the analyses (Experiment 1A: N = 110; Experiment 1B: N = 125; Experiment 2A: N = 121; Experiment 2B: N = 133). In the remaining data, trials with response times below 200 ms or over 3 standard deviations from each participant’s average response times were excluded from the analyses (1.1 to 2.3% of the total trials in each experiment).

#### Stimuli

The competence rating and election judgment tasks used identical set of images of 48 White male politicians from the United States. All original images were freely available from the Internet and all faces were shown in frontal view. Of the 48 candidates, there were 24 pairs of finalists (winners and runners-up) from a total of 18 United States Congressional (16 Senate and 2 House of Representatives), 4 gubernatorial, and 2 mayoral elections that took place between 1996 and 2021. We used a variety of elections mainly due to the limitation that the selected portraits needed to be successfully imported into a software for transformation and not all portraits were easily suitable for the purpose. Note that in the final sample, Democratic candidates won in half of the elections and Republican candidates won in the other half of the elections.

**Figure 1:**
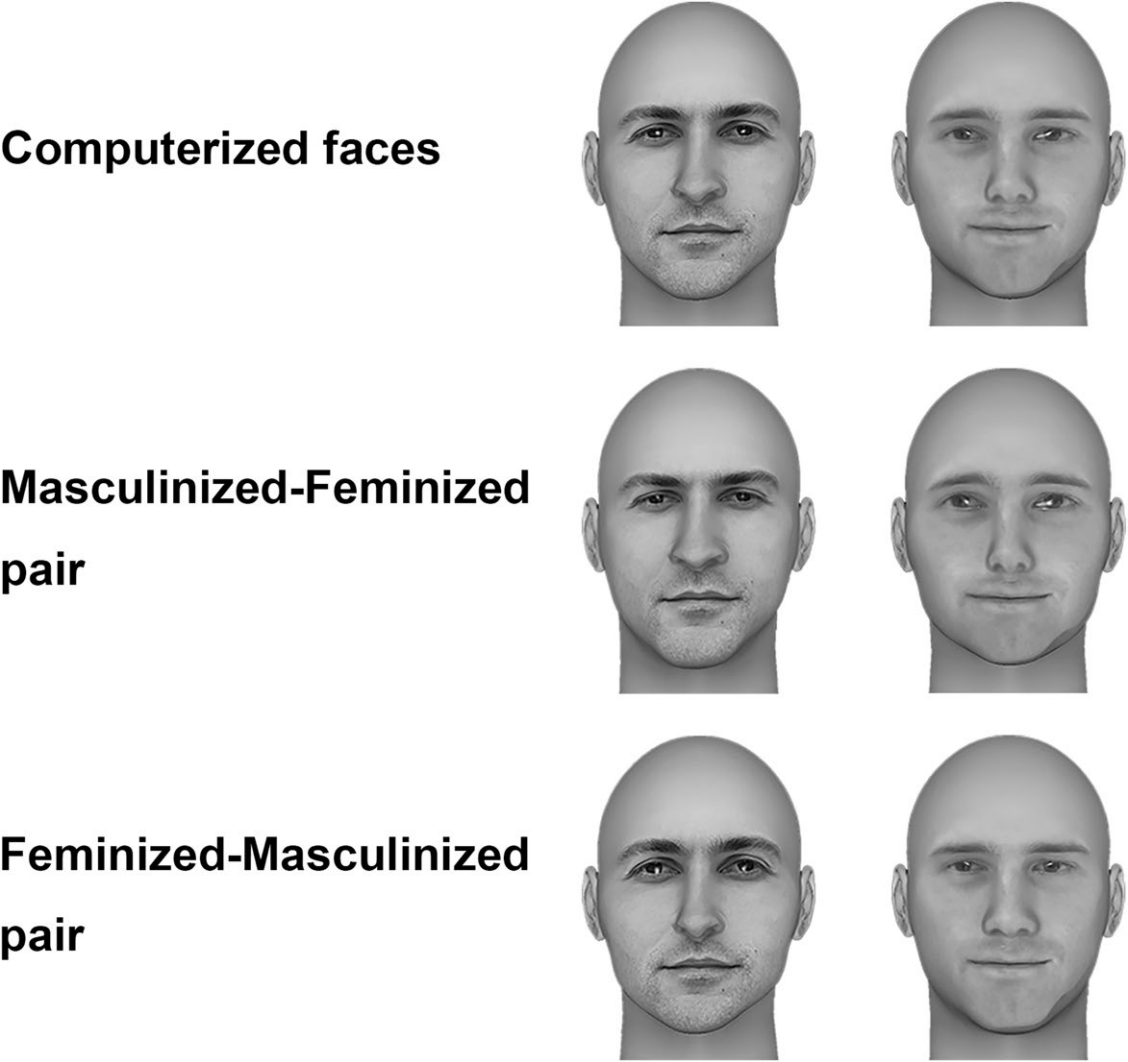
Example images of one pair of faces: computerized faces (Experiment 2), modified faces with masculinized facial features of one face and feminized facial features of another face (Experiments 3 and 4). These faces are for illustration only and were not actual stimuli used in the experiments. Permissions available from the first author.

The images used in Experiment 1 were standardized using Adobe Photoshop, with only the face and a small part of the shoulder kept for each image and any background removed. For the images used in Experiment 2, we used FaceGen Modeller Pro (Singular Inversions, Toronto, Canada) to convert the images used in Experiment 1 into 3D models with a standard face template without hair. The frontal view of the models was then saved for each face. Figure 1 illustrates the sample images of one pair of faces with the transformations used in Experiments 2–4. All images in Experiments 1 and 2 were resized to 284 pixels in height and approximately 220 pixels in width, and were converted to grayscale with luminance equated across the images using the SHINE toolbox^31^.

#### Procedure

Each participant completed two tasks. Apart from the differences in stimuli, the task procedures were identical between Experiments 1 and 2. In the competence rating task, participants rated the perceived competence (“How competent do you think this person is?”) of each of the 48 candidate images once, using a 7-point scale (1: not at all; 7 very much). On each trial, a candidate image was presented for 100 ms. Participants were given unlimited time to respond by a key press. The presentation order of the images was randomized for each participant.

In the election judgment task, participants selected the perceived winner (“Who would win an election?) from each of the 24 pairs of finalists once, using the two response keys corresponding to the faces presented on either the left or right side of the screen. On each trial, each face pair was presented for 1000 ms. Half of the winners were shown on the left and the other half were shown on the right. The presentation order of the face pairs was randomized for each participant.

For both tasks, participants were given practice trials which used similar faces that were not used in the main study. After the completion of both tasks, participants were asked whether they recognized any faces or whether any faces seemed familiar in a 10-choice questions (“Did any of the faces you just saw look familiar to you, or can you name any of them?”). The entire study lasted approximately 8 min.

### Results

**Figure 2:**
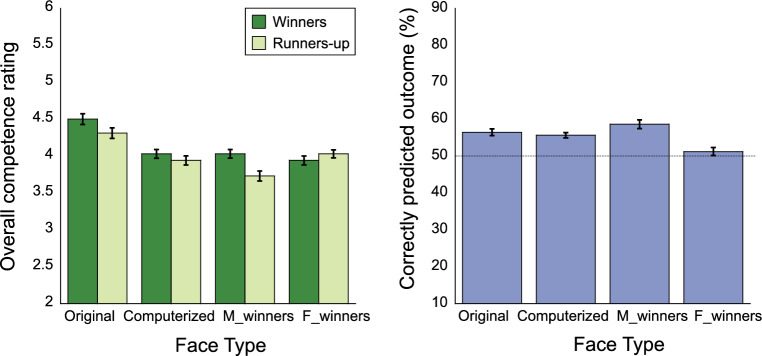
Results from participants who saw the faces for the first time. Left panel: Overall competence ratings for images of the winners and runners-up across the experiments (Experiments 1A, 2A, 3A, and 4A). Right panel: Proportion of correctly predicted outcome across the experiments (Experiments 1B, 2B, 3B, and 4B). M_winners: masculinized winners; F_winners: feminized winners.

**Figure 3:**
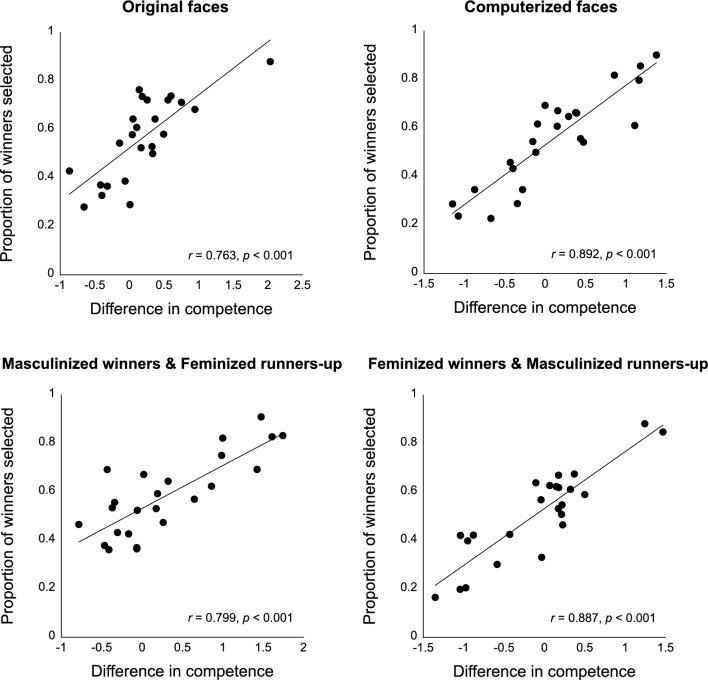
Correlations between the differences in competence ratings between each winner-runner-up pair (ratings from Experiments 1A, 2A, 3A, or 4A) and the proportion of correctly predicted outcome for each pair (prediction accuracy from Experiments 1B, 2B, 3B, or 4B) for original images (Experiment 1), computerized face images (Experiment 2), images of masculinized winners and feminized runners-up (Experiment 3) and images of feminized winners and masculinized runners-up (Experiment 4).

We first report the overall competence ratings and election outcome prediction accuracy in all groups (Experiments 1A, 1B, 2A, and 2B; Table 1). Then we focus on the data when participants saw the images for the first time in each experiment: to examine the role of facial features, we compared the competence ratings between Experiments 1A (original faces; competence judgment first) and 2A (computerized faces; competence judgment first), and the election outcome prediction accuracy between Experiments 1B (original faces; election judgment first) and 2B (computerized faces; election judgment first) (Fig. 2); to examine the relationship between competence evaluation and election outcome prediction, we also report the correlation between the competence ratings and election outcome prediction accuracy for each type of faces (i.e. Experiments 1A: original faces; competence judgment first vs. 1B: original faces; election judgment first; Experiments 2A: computerized faces; competence judgment first vs. 2B: computerized faces; competence judgment first; Fig. 3).

#### Competence ratings within each participant group

We first examined whether the faces of the winners were rated more competent than faces of the runners-up (Table 1). Intraclass correlations of the competence ratings were reported (Table 2), which showed similar results as in previous findings^32^. With the original photographs in Experiment 1, the overall competence ratings were significantly higher for the winners (Experiment 1A: *M* = 4.52, SE = 0.073; Experiment 1B: *M* = 4.51, *SE* = 0.057) than the runners-up (Experiment 1A: *M* = 4.32, SE = 0.072; Experiment 1B: *M* = 4.26, *SE* = 0.055), Experiment 1A: *t*_109_ = 6.68, Cohen’s *d* = 0.637, *p* < 0.001; Experiment 1B: *t*_124_ = 8.45, Cohen’s *d* = 0.756, *p* < 0.001.

**Table 2:** Intraclass correlation coefficients (ICC) of the competence ratings for the images of the political candidates in Experiments 1–4.

Experiment	ICC	95% CI
1A	0.120	0.084–0.180
1B	0.162	0.116–0.236
2A	0.155	0.111–0.226
2B	0.158	0.113–0.231
3A	0.127	0.089–0.189
3B	0.143	0.102–0.211
4A	0.179	0.130–0.258
4B	0.200	0.145–0.284

The ICC estimates and their 95% confident intervals were calculated based on a single-measure, consistency, two-way random effects model.

Importantly, for the computerized faces in Experiment 2, the overall competence ratings were still significantly higher for the winners (Experient 2A: *M* = 4.04, SE = 0.061; Experiment 2B: *M* = 4.23, SE = 0.055) than runners-up (Experiment 2A: *M* = 3.93, SE = 0.064; Experiment 2B: *M* = 4.09, SE = 0.057): Experiment 2A: *t*_120_ = 3.74, Cohen’s *d* = 0.340, *p* < 0.001; Experiment 2B: *t*_132_ = 5.07, Cohen’s *d* = 0.440, *p* < 0.001.

#### Election outcome prediction accuracy within each participant group

We then examined whether the winners were more likely than the runners-up to be selected to win elections. Both Experiments 1 and 2 showed that the percentage of correct guesses for the winners was significantly above chance (50%): Experiment 1A: *M* = 56.4%, SE = 0.90%, *t*_109_ = 7.09, Cohen’s *d* = 0.676, *p* < 0.001; Experiment 1B: *M* = 56.8%, SE = 0.89%, *t*_124_ = 7.58, Cohen’s *d* = 0.678, *p* < 0.001; Experiment 2A: *M* = 55.0%, SE = 0.82%, Cohen’s *d* = 0.555, *t*_120_ = 6.10, *p* < 0.001; Experiment 2B: *M* = 55.7%, SE = 0.75%,* t*_132_ = 7.56, Cohen’s *d* = 0.656, *p* < 0.001. These results suggest that the winners were more likely to be selected to win elections, regardless of whether the original photographs or computerized images were shown.

#### Comparisons between experiments 1A and 2A on overall competence ratings across participant groups who saw the images for the first time

To examine whether the differences in competence ratings between the winners and the runners-up were larger for the original images than the computerized images, a two-way ANOVA with a within-subjects factor (Winning status: winners vs. runners-up) and a between-subjects factor (Experiment 1A: original images; competence judgment first vs. 2A: computerized images; competence judgment first) was conducted on competence ratings. As expected, the significant main effect of Winning status revealed higher competence ratings for winners than runners-up, *F*_1,229_ = 53.67, *η*_p_^2^ = 0.190, *p* < 0.001. The main effect of Experiment was also significant, with higher competence ratings for original images in Experiment 1 than for the computerized images in Experiment 2, *F*_1,229_ = 22.1, *η*_p_^2^ = 0.088, *p* < 0.001. Moreover, the interaction between Winning status and Experiment was significant, *F*_1,229_ = 4.03, *η*_p_^2^ = 0.017, *p* = 0.046, revealing that although the winners were rated more competent than the runners-up in both Experiment 1A (*t*_229_ = 6.45, *p*_tukey_ < 0.001) and Experiment 2A (*t*_229_ = 3.85, *p*_tukey_ < 0.001), a larger difference in competence ratings between winners and runners-up was observed for the original images, compared with that for the computerized images, *t*_229_ = 2.01, Cohen’s *d* = 0.265, *p* = 0.046, presumably due to the additional information available in the original images compared with the computerized images.

#### Comparisons between experiments 1B and 2B on overall election outcome prediction accuracy across participant groups who saw the images for the first time

We also examined whether there was a difference in the election outcome prediction accuracy between the original images and the computerized images. We found that there was no significant difference in election outcome prediction accuracy between the original images (Experiment 1B; election judgment first) and the computerized images (Experiment 2B; election judgment first): *t*_256_ = 0.944, Cohen’s *d* = 0.118, *p* = 0.346.

#### Correlations between competence rating differences and election outcome prediction accuracy

We then examined whether the relative differences in competence ratings between the candidates would positively correlate with the proportion of the winners being selected to win among the pair. Because all participants completed both competence judgment and election outcome prediction tasks, the correlations could be analyzed both within and across participant groups who viewed the same type of faces (i.e. either original faces, or computerized faces). The correlation results within each participant group were reported in Table 1. To highlight the consistency in judgments across participants, we report below the correlations of the two measures across different groups of participants who saw the same type of faces (e.g. original faces) for the first time (Fig. 3). For the original images in Experiment 1, a significant correlation was observed between competence rating differences of the winner-loser pairs in Experiment 1A (original faces; competence judgment first) and election outcome prediction accuracy between the pairs in Experiment 1B (original faces; election judgment first), *r*_22_ = 0.763, *p* < 0.001. For the computerized images in Experiment 2, a similar and significant correlation was also observed between competence rating differences in Experiment 2A (computerized faces; competence judgment first) and election outcome prediction accuracy in Experiment 2B (computerized faces; election judgment first), *r*_22_ = 0.892, *p* < 0.001.

### Discussion

Replicating previous findings on competence judgement from politician photographs^4,6,10,13^, participants who were unfamiliar with the politicians perceived the winning candidates to be more competent than their opponents, based merely on briefly presented images. Although the participants who first completed the competence rating task were not aware of the pairing of the candidates in the actual elections and did not see the candidate pairs simultaneously (i.e. Experiments 1A and 2A), the winners were nonetheless rated to be more competent overall than the runners-up.

Perhaps unsurprisingly, participants generally provided lower competence ratings for the computerized faces (Experiment 2A), compared with the original portraits (Experiment 1A), presumably because the original portraits were more natural in appearance than the computerized faces, and the candidate were all shown in formal suits in the portraits^30^. Nonetheless, although the computerized faces might not have completely captured all aspects of facial characteristics of the candidates, we found that for the computerized faces, the winner faces continued to be rated significantly more competent than the runner-up faces. While the overall difference in competence ratings between the winners and runners-up was larger for the original portraits with additional facial or non-facial information than the computerized face images, these results suggest that facial features are critical in influencing participants’ perceptions of competence of the candidates.

Moreover, participants also performed significantly above chance in predicting the winner from each candidate pair, regardless of whether additional information was present (Experiment 1) or only facial features were present (Experiment 2), and whether participants saw the images for the first time (Experiments 1B and 2B) or the second time (Experiments 1A and 2A). For election outcome prediction, additional information only resulted in numerically, but not statistically significantly, higher accuracy compared with only facial features were present. These results suggest that although competence ratings of individual faces were influenced by the presence of additional information in the original images compared with computerized images, the relative judgment between winners and runners-up was similar for original and computerized images. Participants remained sensitive to the relative differences among the computerized face pairs in evaluating election success between the candidates, suggesting that facial information is critical for the evaluation.

We also found positive correlations between the relative differences in perceived competence between the winners and runners-up and the election outcome prediction accuracy, with the winners who were rated more competent than the runners-up were more likely to be predicted to win the elections. These results were again observed for both the original and computerized image sets, which provided further evidence that perceived competence from facial features is utilized when evaluating likely election outcomes.

To further investigate the effect of facial features on perceived competence and election outcome prediction, we manipulated the facial features of the candidates. Since gender stereotype or typicality has shown to be closely related to perceived competence, in which increased facial masculinity is related to high perceived competence^10,21^, we altered the politician faces by increasing masculinity or femininity of the facial features. In Experiment 3, the winner faces were masculinized and the runner-up faces were feminized. Conversely, the winner faces were feminized and the runner-up faces were masculinized in Experiment 4. Replicating and extending from Experiment 2, we hypothesized that in Experiment 3, the masculinized winners would be perceived as more competent and would be more likely to be predicted as the winners, compared with the femininized runners-up. However, in Experiment 4, the increased masculinity in the runners-up might overcome any initial discrepancies in perceived competence or predicted election success, compared with the winners, especially since the winners were feminized.

Similar to Experiments 1 and 2, all participants in Experiments 3 and 4 completed both the competence rating task and the election judgment task. The presentation order of the two tasks was counter-balanced across participants within each experiment: half of the participants completed the competence rating task prior to the election judgment task (Experiments 3A and 4A); the other half of the participants completed the election judgment task prior to the competence rating task (Experiments 3B and 4B). Likewise, the main analyses between competence rating and election outcome prediction focused on the comparison across participants as they viewed the faces for the first time (e.g. competence ratings in Experiment 3A vs. Experiment 4A; competence ratings in Experiment 3A vs. election outcome prediction in Experiment 3B), but the analyses were also conducted within each participant group (e.g. within Experiment 3A and within Experiment 3B).

## Experiments 3 and 4

### Method

#### Participants

Another group of 600 participants with the same demographics as the participants in Experiments 1 and 2 took part online via the Prolific platform (www.prolific.co): Experiment 3 involved 300 participants and Experiment 4 involved the rest of 300 participants. Within each experiment, 150 participants first completed the competence rating task then the election judgment task (Experiments 3A and 4A) and the rest of the 150 participants first completed the election judgment task then the competence rating task (Experiment 3B and 4B). The study was approved by the New York University Abu Dhabi Institutional Review Board. All participants gave informed written consent prior to the experiment. All methods were performed in accordance with the relevant guidelines and regulations. Following the pre-registered exclusion criteria, data from 498 participants were included in the analyses (Experiment 3A: N = 122; Experiment 3B: N = 126; Experiment 4A: N = 123; Experiment 4B: N = 127). In the remaining data, trials with response times below 200 ms or over 3 standard deviations from each participant’s average response times were excluded from the analyses (1 to 2.2% of the total trials for each experiment).

#### Stimuli and procedure

Using FaceGen Modeller Pro (Singular Inversions, Toronto, Canada), we manipulated the gender characteristics of facial features of the 3D face models from Experiment 2 by either increasing either masculinity or femininity of the faces. The adjustments were based on the ‘face space’ in FaceGen, which was created from a data set consisting of 273 high-resolution 3D face scans of individuals from different gender (60% male and 40% female), age (*M* = 29.71 years old, *SD* = 9.30), and racial groups (approximately 67% European, 11% East Asian, 9% African, 3% South Asian, 10% others), using principal components analysis. Both shape and color information made up the face space. More specifically, there were a total of 80 shape dimensions (50 dimensions of symmetric shape and 30 dimensions of asymmetric shape) and a total of 50 dimensions of symmetric colors. The gender slider is a linear slider aligned with the difference between the average male and the average female, and is scaled such that the male average is valued at − 1 and the female average is valued at + 1. For each face model of the politicians, a masculinized version and a feminized version were created (see Fig. 1). Specifically, the value on the gender slider of each modelled face was decreased by 2 (i.e. − 2) for the masculinized version, and was increased by 2 (i.e. + 2) for the feminized version. Because the values of the masculinized and feminized versions were either below the male average (i.e. − 1) or above the female average (i.e. + 1), masculinity or femininity was made salient in each of the versions. Experiment 3 used masculinized winner faces and feminized runner-up faces. Experiment 4 used feminized winner faces and masculinized runner-up faces. Apart from the facial manipulations, all other aspects of the experiments were identical to those in Experiments 1 and 2.

### Results

We first report the overall competence ratings and election outcome prediction accuracy in all groups (Experiments 3A, 3B, 4A, and 4B; Table 1). We then focus on the data when participants saw the faces for the first time in each experiment: we compared the competence ratings between Experiments 3A (masculinized winners and feminized runners-up; competence judgment first) and 4A (feminized winners and masculinized runners-up; competence judgment first), and the election outcome prediction accuracy between Experiments 3B (masculinized winners and feminized runners-up; election judgment first) and 4B (feminized winners and masculinized runners-up; election judgment first) (Fig. 2). We also report within each experiment the correlation between the competence ratings and election outcome prediction (i.e., for masculinized winners and feminized runners-up, Experiments 3A: competence judgment first vs. 3B: election judgment first; for feminized winners and masculinized runners-up, Experiments 4A: competence judgment first vs. 4B: election judgment first; Fig. 3).

#### Competence ratings within each participant group

We examined whether the faces of the winners were rated more competent than faces of the runners-up in each experiment. Intraclass correlations of the competence ratings were reported in Table 2. For the face images of masculinized winners and feminized runners-up (Experiment 3), the overall competence ratings were significantly higher for the winners (Experiment 3A: *M* = 4.03, SE = 0.058; Experiment 3B: *M* = 4.39, *SE* = 0.051) than the runners-up (Experiment 3A: *M* = 3.73, SE = 0.068; Experiment 3B: *M* = 3.84, *SE* = 0.071), Experiment 3A: *t*_121_ = 5.93, Cohen’s *d* = 0.537, *p* < 0.001; Experiment 3B: *t*_125_ = 9.41, Cohen’s *d* = 0.838, *p* < 0.001.

In contrast, for the face images of feminized winners and masculine runners-up (Experiment 4), different result patterns were observed, with significantly higher overall competence ratings instead for the runners-up (*M* = 4.03, SE = 0.054) compared with the winners (*M* = 3.94, SE = 0.059) in Experiment 4A, *t*_122_ = − 2.27, Cohen’s *d* = − 0.205, *p* = 0.025 and no significant difference between the winners (*M* = 4.06, *SE* = 0.054) and the runners-up (*M* = 4.10, *SE* = 0.053) in Experiment 4B, *t*_126_ = − 0.909, Cohen’s *d* = − 0.0807, *p* = 0.365.

#### Election outcome prediction accuracy within each participant group

We then examined whether the winners were more likely than the runners-up to be selected to win elections. Participants who saw masculinized winners and feminized runners-up performed significantly above chance in predicting the winners in both Experiment 3A (*M* = 64.7%, *SE* = 1.28%), *t*_121_ = 11.5, Cohen’s *d* = 1.041, *p* < 0.001 and Experiment 3B (*M* = 59.3%, *SE* = 1.15%), *t*_125_ = 8.08, Cohen’s *d* = 0.720, *p* < 0.001. However, participants who saw feminized winners and masculinized runners-up performed at chance in predicting the actual winners in both Experiment 4A (*M* = 50.0%, *SE* = 1.13%), *t*_122_ = 0.0427, Cohen’s *d* = 0.004, *p* = 0.483 and Experiment 4B (*M* = 51.2%, *SE* = 1.11%), *t*_126_ = 1.09, Cohen’s *d* = 0.095,* p* = 0.142. These results suggest that an increased masculinity in the winner faces coupled with an increased femininity in the runner-up faces preserved the relatively high prediction accuracy for the winners, whereas an increase femininity in the winner faces coupled with an increased masculinity in the runner-up faces minimized the effect.

#### Comparisons between experiments 3A and 4A for overall competence across participant groups who saw the images for the first time

To examine whether the differences in competence ratings between winners and the runners-up were altered by the masculinity vs. femininity manipulations, a two-way ANOVA with a within-subjects factor (Winning status: winners vs. runners-up) and a between-subjects factor (Experiment: 3A-masculinized winners/feminized runners-up; competence judgment first, and 4A-feminized runners-up/masculinized winners; competence judgment first) on competence ratings revealed a significant main effect of Winning status, with higher competence ratings for winners than runners-up, *F*_1,243_ = 10.4, *η*_p_^2^ = 0.041, *p* = 0.001. The main effect of Experiment was not significant, *F*_1,243_ = 1.84, *η*_p_^2^ = 0.008, *p* = 0.176. Importantly, the significant interaction between Winning status and Experiment, *F*_1,243_ = 36.7, *η*_p_^2^ = 0.131, *p* < 0.001, revealed that winners were rated more competent than runners-up only in Experiment 3A (*t*_243_ = 6.55, *p*_tukey_ < 0.001) but not in Experiment 4A (*t*_243_ = 2.01, *p*_tukey_ <  = 0.188), with a significantly larger difference in competence ratings between masculinized winners and feminized runners-up, compared with that between feminized winners and masculinized runners-up, *t*_243_ = 6.06, Cohen’s *d* = 0.774, *p* < 0.001.

#### Comparisons between experiments 3B and 4B for overall election outcome prediction accuracy across participant groups who saw the images for the first time

When examining whether election outcome prediction accuracy was also affected by the masculinity vs. femininity manipulation, we found a significant difference between the two sets of images used in Experiments 3B and 4B, *t*_251_ = 5.06, Cohen’s *d* = 0.637, *p* < 0.001, with higher election outcome prediction accuracy when the winner faces were masculinized and the runner-up faces were feminized, compared with when the winner faces were feminized and the runner-up faces were masculinized.

#### Correlations between competence rating difference and election outcome prediction accuracy across participant groups who saw the images for the first time

We then examined whether the relative differences in competence ratings between the candidates and the proportion of the winners being selected to win would remain correlated with the masculinized and feminized faces. The correlations conducted within each participant group were reported in Table 1. To highlight the consistency in judgments across participants, the results reported below were the correlations of the two measures across different groups of participants (Fig. 3). Interestingly, significant correlations were observed between competence rating differences of the winner-loser pairs in Experiment 3A (competence judgment first) and election outcome prediction accuracy in Experiment 3B (election judgment first), *r*_22_ = 0.799, *p* < 0.001 and between competence rating differences in Experiment 4A (competence judgment first) and election outcome prediction accuracy in Experiment 4B (election prediction first), *r*_22_ = 0.887, *p* < 0.001, regardless of the masculinity or femininity manipulations on the winner or runner-up faces.

#### Additional comparisons between experiments 3 and 4 with experiment 2

To further evaluate how the gender manipulations affected the competence ratings and election outcome prediction, additional analyses were conducted between Experiment 2 (no gender manipulation) and Experiment 3 (masculinized winners and feminized runners-up), and between Experiment 2 (no gender manipulation) and Experiment 4 (feminized winners and masculinized runners-up). To anticipate the results, for both sets of analyses, the gender manipulations significantly influenced the competence ratings and election outcome prediction between the winners and runners-up.

For competence ratings between Experiments 2A (computerized faces; competence judgment first) and 3A (masculinized winners and feminized runners-up; competence judgment first), there was a significant main effect of Winning status, *F*_1,241_ = 48.96, *η*_p_^2^ = 0.021, *p* < 0.001. The main effect of Experiment was not significant, *F*_1,241_ = 1.60, *η*_p_^2^ = 0.006, *p* = 0.208. Importantly, there was a significant interaction between Winning status and Experiment, *F*_1,241_ = 9.88, *η*_p_^2^ = 0.004, *p* = 0.002: although the winners were rated more competent than the runners-up in both Experiments 2A (*t*_241_ = 2.720, *p*_tukey_ = 0.035) and 3A (*t*_241_ = 7.185, *p*_tukey_ < 0.001), the difference in competence ratings between the winners and runners-up were larger in Experiment 3A than Experiment 2A (*t*_241_ = − 3.14, Cohen’s *d* = − 0.403, *p* = 0.002).

For competence ratings between Experiments 2A (computerized faces; competence judgment first) and 4A (feminized winners and masculinized runners-up; competence judgment first), both main effects were not significant: Winning status, *F*_1,242_ = 0.196, *η*_*p*_^*2*^ = 0.001, *p* = 0.658; Experiments, *F*_1,242_ < 0.001, *η*_p_^2^ < 0.001, *p* = 0.994. However, the significant interaction between Winning status and Experiment, *F*_1,242_ = 16.460, *η*_p_^2^ = 0.064, *p* < 0.001, revealed that the winners were rated more competent than the runners-up in Experiment 2A without the gender manipulation (*t*_242_ = 3.169, *p*_tukey_ = 0.009), but the difference in competence ratings between the winners and runners-up were not statistically significant (*t*_242_ = − 2.566, *p*_tukey_ = 0.053) and the competence ratings for the feminized winners were only numerically lower than the masculinized winners in Experiment 4A.

Likewise, for election outcome prediction, accuracy was lower in Experiment 2B (computerized faces; election prediction first) than Experiment 3B (masculinized winners and feminized runners-up; election prediction first), *t*_257_ = − 2.67, Cohen’s *d* = − 0.332, *p* = 0.008, but was higher in Experiment 2B (computerized faces; election prediction first) than Experiment 4B (feminized winners and masculinized runners-up; election prediction first), *t*_258_ = 3.36, Cohen’s *d* = 0.417, *p* < 0.001.

### Discussion

Across Experiments 3 and 4, we found consistent evidence that gender manipulations on facial features had a strong impact on perceived competence and election outcome prediction of politicians, with the candidates rated more competent with increased facial masculinity and less competent with increased facial femininity. Indeed, as Experiment 2 already showed that the overall perceived difference in competence and election outcome prediction could be based on the existing differences in facial features among the winners and runners-up, Experiment 3 revealed that this advantage for the winners remained robust with the facial features of the winners masculinized and the facial features of the runners-up feminized. In contrast, feminized winners lost such advantage over masculinized runners-up. Instead, these faces appeared to be perceived similarly: Experiment 4 showed no significant difference in perceived competence ratings between the winner and runner-up faces and election outcome prediction performance fell to chance.

As gender manipulations affected judgments of the manipulated faces, it is important to note that the relative judgments among the face pairs remained highly stable, as revealed by the positive correlations observed between perceived competence and prediction outcome accuracy among the candidates in both Experiments 3 and 4. Despite the increased advantage for the winners in Experiment 3 and the diminished advantage for the winners in Experiment 4, facial characteristics that were used to evaluate the individual candidates one group of participants were highly similar to those that were used to predict the winners in another group of participants. The high consensus of these judgments suggests the robustness of the influences of gendered facial characteristics, and not necessarily the existing facial features, on inferred competence.

## General discussion

The goal of this study was to directly investigate the role of facial features of political candidates on perceived competence and election outcome prediction, specifically by direct manipulations of facial features applied to real-world politician faces. Using images of the opposing candidates in real-world elections, we used the original photographs of the candidates, which included additional facial or non-facial features (Experiment 1), and images of the politicians with only facial features extracted (Experiment 2). More importantly, we manipulated the facial features by increasing either masculinity or femininity (Experiments 3 and 4). Participants who had no prior knowledge of the candidates were asked to complete two tasks in a counter-balanced order: either first provided competence ratings based on the briefly presented images of individual candidates, then predicted the winner from each pair of candidates as their images were shown simultaneously, or vice versa.

It is interesting to note that although there might be a subtle difference in asking participants to cast a hypothetical vote (i.e. “Who would you vote for?”)^4,6^, compared with asking participants to predict or guess who would win or who won, it has been shown that either question led to similar results^3^. Indeed, Experiment 1 extended and replicated previous findings^3,4,6^ that overall, the winners in the elections were perceived to be more competent and were more likely to be predicted to win the elections, compared with the runners-up. Critically, although it is possible that the computerized images might not completely capture all subtle facial characteristics, Experiment 2 showed that the results from Experiment 1 were at least partially due to the perceptions and interpretations of the politicians’ facial features, since the same result patterns were found for the computerized images of the politician faces, when additional information including some facial features and all non-facial features were removed. By systematically comparing the results for the original photographs and those for the computerized facial images, we found that the influence of additional facial or non-facial features from the original images was indeed present in competence evaluation, as participants also utilized any information such as clothing to shape impressions^30,33^, when it was available to form their judgments. Although the differences in competence ratings between the winners and the runners-up were larger for the original than computerized images, it is important to note that the comparable election outcome prediction accuracy between the original and computerized faces suggests that the relative differences between the winners and runners-up in the original images persisted in the computerized images. The current findings using computerized real-world politician faces provide direct evidence that facial features alone are informative for naïve participants to quickly form judgments about the politicians and to correctly predict the actual election results better than chance.

More importantly, the present study provided an experimental manipulation to reveal the influence of facial features on both competence and election prediction judgments at a first glance. When gender manipulations were directly applied to the same real-world politician faces in Experiments 3 and 4, we found that the relatively subtle manipulations of facial features can have robust effects on perceived competence and predicted election outcome. Note that since the original facial features largely remain, the changes of the features along the masculinity-femininity continuum were likely only obvious when the different versions of the faces were shown together (Fig. 1). Consistent with previous findings that masculinity is often associated with competence and competent-looking individuals are more likely to be perceived as men than women^10,21^, we found that masculinized facial features further enhanced the winners’ advantage in perceived competence and predicted winning outcome over the feminized runners-up. In contrast, by feminizing the winner faces and masculinizing the runner-up faces, any existing differences between the faces appeared to have been minimized, resulting in a lack of difference in perceived competence or predicted election success for the faces. Together, these findings demonstrated that the initial differences between the faces of the winners and runners-up indeed existed, and that the impressions could be changed by increasing or decreasing gendered facial characteristics, which may be an over-generalization from the common gender stereotypes for leaders^34^.

It is also important to note that although the winner faces generally appeared to implicate competence or leadership quality, there were relative differences among different face pairs: some winners were rated much more competent than their runners-up while some less so, and some runners-up were rated more competent than their opponents instead (Fig. 3). Although all participants in this study were unfamiliar with the politicians, the relative difference in perceived competence and the election outcome prediction accuracy among candidate pairs was highly correlated in all experiments. Specifically, the politician faces that were consistently rated to be more competent were also more likely to be selected as the winners of the elections, regardless of the final election outcomes. Such correlation was observed both within and across participants (Table 1; Fig. 3). Notably, although Experiments 3 and 4 showed that perceptions and interpretations of personality traits of individual faces could be affected by gendered facial manipulations, the high consensus suggests that similar types of diagnostic facial information were used for evaluating competence and selecting the prospective winners among participants.

What are the diagnostic facial features for perceiving competence? While it is possible to quantify different levels of perceived competence on faces using data-driven approaches^25–27^, it remains difficult to detail the specific types of facial features that implicate an individual’s level of competence. Consistent with previous studies^10,21^, our results provide further support on the role of masculinity. Nonetheless, a potential limitation is that the current study examined this question using faces of White male politicians only. We are certainly aware of the potential issues in generalizing the findings to non-White male samples^35,36^, but at the moment, it is unlikely feasible to collect a sufficiently large face image set of politicians from a relatively diverse backgrounds (e.g. female) with matched qualities for the two final candidates in an election (e.g. both the winner and runner-up were female). Indeed, there have only been a relatively small number of previous elections with the two final candidates being either both female or both of another gender. Therefore, it remains unclear whether the role of facial masculinity may also benefit non-male individuals, as facial sex typicality may influence perceived competence and vote choices for politicians^20,22,23,37,38^. Moreover, it is possible that the relationship between masculinity and leadership quality is a social construct, instead of a biological one. With increasing diversity in the demographics of politicians or individuals in leadership roles^34^, future research may investigate how such changes would affect the stereotype of leaders and how different types of facial features might be instead used to infer competence or leadership quality from faces.

## Conclusion

Although previous research has consistently reported the importance of visual facial cues of political candidates in predicting election outcomes, most studies used candidate photographs which also included additional facial or non-facial cues. By isolating and manipulating the facial features, this study provided direct evidence that facial features alone were used by observers to interpret other individuals’ personality traits or leadership ability. These findings are critical in constructing hypotheses on how different types of visual information^39^, and non-visual information, may be utilized for choosing leaders.

## Data Availability

The dataset and analysis files are available on https://osf.io/ykpmf/.
